# Comparison of foods and beverages served and consumed in Child and Adult Care Food Program-participating childcare centres to national guidelines

**DOI:** 10.1017/S136898002300109X

**Published:** 2023-09

**Authors:** Rebecca S Mozaffarian, Sonia Carter, Mary C Bovenzi, Erica L Kenney

**Affiliations:** 1 Department of Nutrition, Harvard TH Chan School of Public Health, 665 Huntington Ave, Boston, MA 02115, USA; 2 Boston Public Health Commission, Boston, MA 02118, USA; 3 Department of Social and Behavioral Sciences, Harvard TH Chan School of Public Health, Boston, MA 02115, USA

**Keywords:** Childcare, CACFP, Dietary guidelines, Nutrition, Health

## Abstract

**Objective:**

The federal Child and Adult Care Food Program (CACFP) sets minimum nutrition and portion size standards for meals served in participating childcare programs. CACFP has been associated with more nutritious meals served. It is unclear, however, whether CACFP results in children’s dietary intake being aligned with national recommendations. We assess whether children’s dietary intake in CACFP-participating childcare centres meets benchmarks set by the Dietary Guidelines for Americans (DGA).

**Design:**

This is a cross-sectional study. We used direct observation to estimate quantities of foods/beverages served and consumed per child. Mean amounts served per child per day were compared with CACFP portion size requirements for each component (fruits, vegetables, milk and meat/meat alternate). Mean amounts of foods/beverages consumed were compared with DGA recommendations (energy content, fruits, vegetables, whole/refined grains, dairy, protein and added sugars). One sample t-tests evaluated if quantities served and consumed were different from CACFP and DGA standards, respectively.

**Setting:**

Six CACFP-participating childcare centres.

**Participants:**

2–5 year-old children attending childcare.

**Results:**

We observed forty-six children across 166 child meals. Most meals served met CACFP nutrition standards. Compared with CACFP portion size standards, children were served more grains at breakfast and lunch; more fruits/vegetables at lunch but less at breakfast and snack and less dairy at all eating occasions. Compared with DGA recommendations, children under-consumed every food/beverage category except grains during at least one eating occasion.

**Conclusions:**

Children were served quantities of foods/beverages mostly consistent with CACFP portion size requirements, but had sub-optimal intake relative to DGA. More research is needed to help children consume healthy diets in childcare.

Early childhood is a critical time for habit formation related to behaviours that contribute to healthy weight and reduced risk of disease^(1,2)^. A quarter of preschool-age children in the United States have overweight or obesity^(3)^, and more have suboptimal dietary habits^(4,5)^. Over 60 % of preschool-age children attend regular childcare, and among these children approximately 80 % attend centre-based care. Childcare programmes have the potential to meaningfully impact the quality of children’s diets by offering nutritious meals and snacks^(6–8)^. Yet, children attending childcare programmes under-consume recommended energy content and health-promoting food groups (e.g. fruit, vegetables and whole grains) when compared with dietary benchmarks^(9–13)^.

The Child and Adult Care Food Program (CACFP) is a federal food assistance program that provides reimbursable meals and snacks to early childcare programmes serving low-income families that opt into the programme. To be eligible to participate in CACFP, childcare centres must provide meals and either have nonprofit status or serve a certain percentage of children from families with low incomes. CACFP reaches over 4 million children annually in childcare and provides reimbursements for meals and snacks served at childcare that meet a set of minimum nutrition standards. Foods and beverages provided to children in CACFP programs must meet portion size requirements and nutrition standards to be reimbursed^(14)^. As required by the Healthy, Hunger-Free Kids Act, in 2017 the United States Department of Agriculture released new CACFP regulations to better align the nutrition standards with the 2015–2020 Dietary Guidelines for Americans (DGA), which serve as U.S. national guidelines for optimal dietary intake^(15–18)^. (see online supplementary material, Supplemental Appendix 1). CACFP-participating programmes usually have healthier meal service than non-participating programmes^(19–25)^, and children attending such programmes may have better dietary intake than children in non-participating programmes^(26,27)^.

While CACFP specifies requirements for meals that are intended to support healthy development and adequate intake, they are guidelines for food service, and not individual-level intake, as the DGA are^(15)^. There are no specific DGA recommendations for the amount children should consume during childcare, but it has been recommended by researchers that full day childcare centres should provide children with 50–67 % of their daily required intake through breakfast, lunch and one snack^(28,29)^.

There have been few studies evaluating the amount of food and beverages consumed in CACFP-participating childcare programmes specifically, and how they compare to DGA, which was the guiding document that informed the updated CACFP standards. One study from Nebraska found that foods and beverages served at lunch only in CACFP-participating centres met CACFP guidelines, but that children’s actual dietary intake did not reflect recommended levels of intake of different foods and beverages in the DGA^(29)^.

We aimed to assess whether the 2017 updates to the CACFP meal patterns resulted in children’s dietary intake being aligned with the DGA at breakfast, lunch and snack during childcare. Using direct observation methods to measure intake among a racially/ethnically diverse sample of children, and observing across several meals and two days, we evaluated how much children’s intake of CACFP meals and snacks differed from DGA recommendations in CACFP-participating centres in Boston.

## Methods

### Study design and population

Childcare programmes were eligible to participate in this cross-sectional study if they were a licensed centre-based facility in Boston and participated in CACFP. Children were eligible if they attended a participating programme and were aged 2–5 years old. Our project goal was to recruit approximately ten children from sixteen early childcare and education programmes (160 children total). However, due to programme closures and safety precautions related to the COVID-19 pandemic, we had to end recruitment and observations early. Using a publicly available website maintained by the MA Department of Early Education and Care, we downloaded a list of 133 licensed childcare centres located in Boston and recruited study participants between September and December 2019^(30)^. Study staff contacted childcare directors by email and phone to verify eligibility criteria and invite them to participate in the study. Fifty-five (41·4 %) were excluded because they did not participate in CACFP. Of the seventy-eight remaining programmes; 47 (60·3 %) did not respond after the researchers’ third contact attempt and were considered non-responders; 12 (15·4 %) declined to participate; 7 (9·0 %) agreed to participate in an interview only (did not agree to participate in data collection); 4 (5·1 %) had no available contact information and 8 (10·3 %) agreed to participate. After agreeing to participate, one programme closed due to the COVID-19 safety precautions prior to collecting any data, and another programme was later excluded after it was ascertained that they did not actually participate in CACFP. For this analysis, we include the results from six childcare programmes. We collected 2 d of dietary intake data from four centres, and 1 d from two centres whose data collection period was scheduled shortly before childcare programmes unexpectedly closed due to COVID-19 restrictions in March 2020, precluding us from returning to the programme for a second visit.

### Measures

#### Observation of dietary intake in childcare centres

From January–March 2020, researchers conducted two non-consecutive weekdays of direct dietary intake observation. Prior to observations, research assistants received training to visually assess serving sizes of foods and beverages served and consumed using the Direct Observation in Childcare protocol^(31)^. Data collection was scheduled on two non-consecutive weekdays. Each research assistant was assigned to observe up to three participating children during breakfast, lunch and snack. They quantified the amounts of foods and beverages children were served and consumed during each eating occasion followed by the amount remaining at the end of the eating occasion, as well as amounts spilled and additional helpings^(27,31,32)^.

To calculate the quantity of foods and beverages consumed per eating occasion per child, we subtracted the recorded serving sizes at the end of each eating occasion from the amount served at the beginning. Additional helpings (taken by 13·9 % of children) were included in the amount served, and spills (1·8 % of all observed food and beverage items taken per child) were subtracted from the amount left at the end.

The research team created a database of nutrient information per standard serving sizes for each item observed (*n* 83 unique foods and beverages) using Nutrition Facts and ingredients labels or recorded information from product manufacturer’s website for packaged foods. For items served without a nutrition label, we recorded the nutrient content from the U.S. Department of Agriculture Food Data Central Database^(33)^. This enables accurate analysis of brand-specific nutrient information and has been used to assess energy and nutrient content of foods and beverages served and consumed by children in previously published studies^(34–36)^. To calculate the energy content and servings of food and beverage categories, we multiplied the estimated serving size served and consumed by the nutrient information for the corresponding food or beverage. We calculated total amount served and consumed per eating occasion by summing the amount for each category.

#### Alignment of foods and beverages served with CACFP standards and consumed with DGA recommendations

CACFP specifies food service requirements for meals that are intended to support healthy growth, not individual-level intake; the programme does not specify what children should actually consume. The DGA are guidelines for optimal individual dietary intake^(15)^, and CACFP’s meal pattern standards are ostensibly meant to be aligned with the DGA. The DGA, however, are not meant to be guidelines for what childcare programmes can served. Therefore, we evaluated the foods *served* by comparing to the CACFP meal pattern standards and the foods *consumed* by comparing to the DGA. While the DGA do not specify how much children should consume in childcare settings, researchers recommend that full-day childcare centres should provide 50 % – 67 % of children’s daily required intake through breakfast, lunch and one snack^(28,29)^.

To determine if foods and beverages served to children were meeting CACFP minimum standards, we compared the amounts served of each CACFP major food category (fruits/vegetables, milk, grains and meat/meat alternates) with the age-specific minimum required servings for each meal/snack^(18,29)^. We also estimated adherence to the 2017 updated nutrition standards by assessing whether (1) a fruit and vegetable or two vegetables were served at lunch; (2) whole grains were served at least once per day; (3) yogurts served had no more than 23 g of sugar per 6 oz servings; (4) cereals served had no more than 6 g sugar per oz; (5) 100 % juice was limited to one 4 oz serving per day and (6) grain-based desserts were not served (see online supplementary material, Supplemental Appendix 1).

To then determine if children’s actual intake from these CACFP meals was meeting the DGA, we first assumed that 50–67 % of children’s recommended daily intake would be in childcare, with one-third (25–33·5 %) provided at lunch and the remaining one-third provided at breakfast and snack (one-sixth each; 12·5–16·8 %)^(10,21,28,29,37)^. We then calibrated benchmarks for total consumption and by meal/snack type by applying these proportions to DGA age and sex-specific standards for daily intake (e.g. 1000 kcal per day for a moderately active 2 year old girl or boy; 1200 kcal per day for a moderately active 3 year old girl; and 1400 kcal for a moderately active 4–5 year old girl or boy)^(15,29,38)^. Ten children (7·7 %) did not report their ages; for these children we applied the DGA standards for the median age in our sample (3 years old). To determine intake relative to estimated DGA recommendations, proteins and grains (including refined and whole grains separately) were converted into ounce equivalents; fruits, vegetables and dairy (including milks and dairy foods) were converted into cup equivalents. See online supplementary material, Supplemental Appendix 2 for a detailed description of DGA serving sizes and thresholds for each age and sex group by food category and eating occasion.

### Statistical analysis

We calculated frequencies and means for programme and child-level demographics. We calculated the mean energy content and amounts of foods and beverages served and consumed per child per meal, adjusted for clustering of observations within children and children within centres.

To quantify differences between the amounts of foods and beverages served with the minimum CACFP serving size guidelines, we subtracted the amounts served from the age-based guidelines for milk, fruits/vegetables, grains and meat/meat alternates for each meal and snack, creating difference scores. Similarly, we subtracted the amounts consumed for each child at each meal/snack for energy content, fruits, vegetables, grain (refined and whole), dairy and protein from age and sex-based DGA recommendations (scaled to each meal/snack as described above). We used one-sample t-tests to then assess whether these differences – i.e. in servings from CACFP requirements, and in intake from DGA recommendations – were statistically significantly different from zero.

Lastly, we explored if the amounts of foods and beverages served would have supported DGA intake recommendations if children had fully consumed what they were served. For this analysis, we subtracted the amount *served* (not consumed) from the DGA recommendations and used one-sample *t* test to assess differences as above. Because of the multiple comparisons made in the study, we used a Bonferroni correction, setting alpha < 0·001. All analyses were performed using SAS version 9.4 (SAS Institute).

## Results

Across the six centres (one classroom per centre), we observed forty-six children consuming food and beverages over 166 child-meals, including breakfast (*n* 42, 25 %), lunch (*n* 73, 44 %) and afternoon snack (*n* 51, 31 %). Two centres were independent programmes that prepared meals on-site, and four were sponsored centres that had vendors providing meals. Almost all programmes served meals as prefixed portions or portioned by staff, and on very few occasions meals/snacks were served as both pre-fixed portions/portioned by staff and family style service (one programme for snack on one day of observation; one programme for breakfast/lunch/snack on both days of observation). Thirty-seven percent of the children observed were Black, 46 % were male and the mean age was 3·3 years old (sd ± 0·9) (Table 1).

**Table 1:** Characteristics of childcare programs, meals and snacks and children observed in 6 early childcare centres participating in CACFP, Boston December 2019–March 2020*

	Number	(%)
Childcare program-level observations		
*n* programs observed	6	
Sponsorship status		
Independent/on-site prep	2	33·3
Sponsored/vendor	4	66·7
*n* observation days	10	
*n* eating occasions observed	25	
Breakfast	7	28·0
Lunch	10	40·0
Snack	8	32·0
Child level observations		
*n* children observed	46	
*n* child days observed	77	
*n* child-meals observed	166	
Breakfast	42	25·3
Lunch	73	44·0
Snack	51	30·7
Child demographics		
Age, years		
Mean (±)	3·3	
sd	0·9	
Sex, *n* male†	20	45·5
Race/Ethnicity‡		
White	7	18·4
Black	14	36·8
Hispanic	6	15·8
Asian	6	15·8
Other/Mixed	5	13·2

* See Appendix 2 for the full Dietary Guideline for Americans recommended ranges for energy content and food group by age and sex.

† *n* 44.

‡ *n* 38.

Among the meals served, 71 % of breakfasts, 70 % of lunches and 88 % of snacks met the CACFP nutrition and best practice standards that had been introduced in 2017. All meals and snacks included whole grains daily and limited juice, and 90 % did not include grain-based desserts or sugary beverages. All yogurts and cereals served met sugar standards. Milk was not served at three of the ten lunches observed (see online supplementary material, Supplemental Appendix 3).

Compared with CACFP’s minimum portion size requirements, children were served more grains (0·41 oz, s e ± 0·10) and fewer fruits (–0·15 c, se ± 0·04) at breakfast and more grains (3·45 oz, se ± 0·36), fruits (0·44 c, se ± 0·08) and vegetables (0·18 c, se ± 0·04) at lunch. Children were served fewer fruits (–0·31c, se ± 0·04) and vegetables (–0·46 c, se ± 0·02) at snack. Children were served less skim/1 % milk at breakfast (–0·26 c, se ± 0·04), lunch (–0·43 c, se ± 0·03) and snack (–0·46 c, se ± 0·01) than the minimum portion size requirements. (*P* < 0·001 each, Table 2)

**Table 2:** Child and adult care food program minimum nutrition and portion size standards and mean differences (±se) between the amount of foods and beverages served among preschool-aged children attending breakfast, lunch and/or snack during childcare. Boston MA, December 2019–March 2020. (*n* 166 child-meals)*,†,‡

	CACFP portion size child age 2 years	CACFP portion size child age 3–5 years	Amount served (cups or ounces)				Differences between foods and beverages served from recommended amounts (accounting for age/sex)		
			*n*	% observations	Mean	se	Mean difference	se	*P* value
Breakfast (*n* 42)									
Fruit/vegetable (c)	0·25	0·50	37	88·1	0·24	0·03	−0·15	0·04	0·0005
Grain (oz)	0·50	0·50	35	83·0	0·91	0·09	0·41	0·10	0·0002
Skim/1 % white milk (c)	0·50	0·75	34	81·0	0·38	0·05	−0·26	0·04	< 0·0001
Meat/meat alternates (oz)	–	–	7	16·7	0·96	0·36	–	–	–
Lunch (*n* 73)									
Fruit (c)	0·13	0·25	56	76·7	0·67	0·09	0·44	0·08	< 0·0001
Vegetable (c)	0·13	0·25	72	98·6	0·40	0·05	0·18	0·04	< 0·0001
Grain (oz)	0·50	0·50	66	90·4	3·97	0·35	3·45	0·36	< 0·0001
Skim or 1 % white milk (c)	0·50	0·75	42	57·5	0·26	0·03	−0·43	0·03	< 0·0001
Meat/ meat alternates (oz)	1·00	1·50	41	56·2	1·41	0·13	−0·03	0·16	0·86
Snack (*n* 51)									
Fruit (c)	0·50	0·50	21	41·2	0·19	0·02	−0·31	0·04	< 0·0001
Vegetable (c)	0·50	0·50	7	13·7	0·04	0·02	−0·46	0·02	< 0·0001
Grain (oz)	0·50	0·50	42	82·4	0·75	0·09	0·25	0·14	0·08
Skim or 1 % white milk (c)	0·50	0·50	9	17·7	0·04	0·01	−0·46	0·01	< 0·0001
Meat/meat alternates (oz)	0·50	0·50	25	49·0	0·93	0·15	0·43	0·20	0·04

* Reference: United States Department of Agriculture. Updated Child and Adult Care Food Program Meal Patterns: Child and Adult Meals. https://fns-prod.azureedge.net/sites/default/files/cacfp/CACFP_MealBP.pdf accessed 11/09/2021.

† Meat and meat alternates may be used to substitute the entire grains component a maximum of three times per week at breakfast. All five components required for a reimbursable meal at lunch; two vegetables can be offered at lunch in lieu of one fruit and one vegetable. Select two of the five components for snack.

‡ Adjusted for clustering of observations within children and children within centers.

Children consumed fewer vegetables at breakfast (–0·19 se ± 0·01), lunch (–0·23 se ± 0·04) and snack (–0·20 se ± 0·004) compared with DGA recommendations (*P* < 0·0001 each). Children consumed less dairy than recommended at lunch (–0·46 se ± 0·03) and snack (–0·26 se ± 0·02). Children consumed less protein (–0·33 oz, se ± 0·07) than recommended at breakfast; fewer energy content (–362 kJ se ± 97·1) but more grains (1·38 oz, se ± 0·33) at lunch and fewer whole grains (–0·23 oz, se ± 0·03) and protein (–0·33 oz, se ± 0·07) than recommended at snack. (*P* < 0·001 each, Table 3)

**Table 3:** Mean differences (±se) between estimated Dietary Guidelines for Americans recommendations and amount of foods and beverages served and consumed among preschool-aged children attending breakfast, lunch and/or snack during childcare. Boston MA, December 2019–March 2020. (*n* 166 child-meals)*,†,‡

	Mean amount consumed (kJ, cups or oz)		Differences between foods and beverages consumed from recommended amounts (accounting for age/sex)			Mean amount served (kJ, cups, or oz)		Differences between foods and beverages served from recommended amounts (accounting for age/sex)		
	Mean	se	Mean difference	se	*P* value	Mean	se	Mean difference	se	*P* value
Breakfast (*n* 42)										
Energy content (kJ)	589	21·2	−131·0	45·0	0·006	833	27·0	113·2	50·7	0·03
Fruit (c)	0·20	0·03	0·03	0·03	0·43	0·23	0·03	0·07	0·03	0·05
Vegetable (c)	0·00	0·00	−0·19	0·01	<0·0001	0·00	0·00	−0·19	0·01	<0·0001
Grain (oz)	0·57	0·07	−0·002	0·09	0·97	0·91	0·09	0·34	0·10	0·003
Whole grains (oz)	0·24	0·06	−0·04	0·06	0·53	0·37	0·08	0·09	0·08	0·33
Refined grains (oz)	0·32	0·05	0·04	0·09	0·66	0·53	0·07	0·25	0·11	0·03
Dairy (c)	0·42	0·05	0·08	0·05	0·11	0·52	0·06	0·18	0·05	0·0005
Protein (oz)	0·09	0·07	−0·33	0·07	<0·0001	0·20	0·14	−0·22	0·14	0·13
Lunch (*n* 73)										
Energy content (kJ)	1193	95·5	−361·9	97·1	0·0004	1841	90·9	285	108·2	0·01
Fruit (c)	0·40	0·05	0·02	0·06	0·74	0·67	0·09	0·29	0·08	0·0003
Vegetable (c)	0·18	0·04	−0·23	0·04	<0·0001	0·40	0·05	−0·007	0·04	0·85
Grain (oz)	2·65	0·31	1·38	0·33	<0·0001	3·97	0·35	2·67	0·38	<0·0001
Whole grains (oz)	1·08	0·16	0·44	0·18	0·02	1·51	0·22	0·87	0·22	0·0002
Refined grains (oz)	1·55	0·33	0·91	0·35	0·01	2·46	0·440	1·80	0·44	<0·0001
Dairy (c)	0·24	0·02	−0·46	0·03	<0·0001	0·28	0·03	−0·41	0·03	<0·0001
Protein (oz)	0·98	0·13	0·01	0·14	0·96	1·43	0·13	0·42	0·16	0·01
Snack (*n* 51)										
Energy content (kJ)	531	82·6	−263·6	90·8	0·006	747·5	90·2	−46·7	93·7	0·62
Fruit (c)	0·11	0·02	−0·09	0·03	0·003	0·19	0·02	−0·01	0·04	0·74
Vegetable (c)	0·00	0·00	−0·20	0·004	<0·0001	0·04	0·02	−0·16	0·02	<0·0001
Grain (oz)	0·57	0·09	−0·09	0·14	0·53	0·75	0·09	0·09	0·14	0·51
Whole grains (oz)	0·10	0·02	−0·23	0·03	<0·0001	0·13	0·02	−0·20	0·03	<0·0001
Refined grains (oz)	0·47	0·07	0·14	0·14	0·33	0·62	0·08	0·29	0·15	0·06
Dairy (c)	0·09	0·01	−0·26	0·02	<0·0001	0·12	0·01	−0·23	0·02	<0·0001
Protein (oz)	0·18	0·05	−0·33	0·07	<0·0001	0·28	0·06	−0·23	0·07	0·003

* Median Dietary Guideline recommendations for moderately active female children age 3 years old consuming breakfast, lunch and snack during childcare are as follows: 702 kcal, 0·6 cups fruit, 0·9 cups vegetables, 2·3 oz grain (1·2 oz each whole grains and refined grains), 1·5 c dairy, 1·8 oz protein. For all values used to determine if children are meeting Dietary Guidelines recommendations by age and sex, see online supplementary material, Supplemental Appendix 2.

† Ten children (7·7 %) did not report their ages, thus we applied the DGA standards for the median age in our sample (3 years old).

‡ Added sugars were within the recommended limits of < 10 % of total energy content at all meals.

Lastly, we evaluated whether DGA targets would have been met if children had consumed all meals and snacks served, to assess whether CACFP servings would have resulted in DGA adherence under ideal conditions. Even if children had consumed all foods and beverages served at each meal and snack, they would have consumed more dairy than recommended at breakfast (0·18 c, se ± 0·05); less dairy than recommended at lunch (–0·41 oz, se ± 0·03) and snack (–0·23 oz, se ± 0·02) and fewer vegetables (–0·16 c, se ± 0·02) and whole grains (–0·20 oz, se ± 0·3) at snack (*P* < 0·0001 each, Table 3). Children would have exceeded DGA targets at lunch for fruit (0·29 c, se ± 0·08) and grains (total 2·67 oz, se ± 0·38; whole grains 0·87 oz, se ± 0·22; refined grains 1·80 oz, se ± 0·44) if they had consumed all foods served to them (*P* < 0·0001 each, Table 3)

## Discussion

In this study, we quantified what children are served and consume while attending breakfast, lunch and snack at CACFP-participating childcare programmes in Boston. Our findings provide a unique addition to the literature by collecting dietary data across breakfast, lunch and snack (not only lunch) in a racially/ethnic diverse group of preschool children and comparing the results to national guidelines for both servings and consumption.

We found that meals and snacks served at childcare centres during data collection were compliant with the CACFP minimum nutrition standards for the majority of meals and snacks observed. At lunch, children were served more than recommended CACFP portion sizes of fruit, vegetables and grains, suggesting that CACFP meal pattern and minimum portion size requirements are largely being met during the lunch period. However, providers in this sample were not as compliant with the requirement to serve milk at lunch as has been observed in prior studies of CACFP-participating programmes^(29,32,36,39–41)^; as milk was not served at three out of the 10 lunches observed (see online supplementary material, Supplemental Appendix 3). It is unclear why the providers in our sample did not meet this requirement. Meanwhile, providers predominantly met the newer CACFP nutrition standards by rarely serving sugary beverages, grain-based desserts or juice more than once per day. All programmes only served yogurt and cereals that met sugar standards.

However, even though the breakfasts, lunches and snacks observed were compliant with CACFP, most 2–5-year-0old children consuming these meals and snacks were nonetheless not meeting DGA recommendations. With the exception of total and refined grains, every food category was significantly under-consumed during at least one meal or snack. Compared with the estimated recommended DGA intake, children were consuming significantly fewer vegetables at breakfast, lunch and snack; less dairy at lunch and snack; less protein at breakfast and snack; fewer energy content at lunch; fewer whole grains at snack and more grains at lunch. An encouraging finding was that children were served and consumed added sugars in amounts that were below the DGA’s recommended limits of 10 % of total energy, suggesting that CACFP meals may be lower in added sugars compared with meals that children consume elsewhere^(42,43)^. Our findings are consistent with other studies observing lunch consumption during the childcare day^(29)^, as well as national studies analysing diet quality and adherence to the DGA among young children^(4)^.

Although the 2017 updates to the CACFP meal patterns (e.g. servings of whole grains, limits to 100 % juice and limits to the sugar content of cereals and yogurts) represented an important update in CACFP guidance and improvements in food service in some centres^(23,40)^, our results suggest that the updated standards may not have gone far enough to bring children’s dietary intake in line with DGA recommendations. However, despite our findings that children’s dietary intake at childcare could be further improved, previous studies have indicated that children attending childcare consume healthier diets while in care compared to when they are not^(42,44)^. Thus, although there is still room for improvement, childcare is an important setting to where healthy eating behaviours are established in young children.

It is possible that the voluntary ‘best practice’ standards in CACFP – which specify an additional set of slightly stronger nutrition standards that are more in line with recommendations from the National Academy of Medicine than the existing minimum nutrition standards – could have helped bring children’s intake more in line with the DGA. However, providers may need more support in developing menus and processes that would promote healthier eating. While trainings and technical assistance for providers could potentially help, participating in such trainings is a time commitment, and the standards can still be confusing for programmes to follow, particularly for independent centres that do not have a sponsoring agency which might be able to pre-plan menus and handle food ordering centrally^(25,45,46)^. For centres that do work with sponsors and purchase their foods/beverages from a food service vendor, the vendors themselves, in partnership with nutritionists on staff at sponsoring agencies, often take responsibility for ensuring that the meals they provide meet reimbursable CACFP standards, thus relieving centre directors of this task and time commitment. Supporting independent centres by providing access to nutritionists and potentially food service vendors who can ensure meals meet stronger standards could be a strategy to improve the quality of meals served without increasing childcare provider burden.

Our study highlights that more research is needed to evaluate why children under-consume the foods offered to them in childcare settings. Our study is not the first to show that children consume less than recommend amounts of energy content and health-promoting foods in childcare^(27,32,47)^. Yet the reason for under-consumption may not be that CACFP guidelines alone are inadequate. In this study, children were mostly served the appropriate amounts of food to meet DGA recommendations at breakfast and lunch, suggesting other factors, such as the appeal and palatability of the foods offered, might be at play. At snack, however, inadequate quantities of vegetables and whole grains were served, suggesting that existing CACFP standards may not be strong enough to support children in meeting DGA at snack. Taken together, our findings suggest more research is needed to not only identify the best strategies and standards for food service for children but also to identify potential barriers to full consumption of meals served in CACFP-participating childcare centres.

### Strengths and limitations

Strengths of our study include using direct, validated observation measures in a racially/ethnically diverse sample of children. Our study also measured what was served and consumed across multiple meals and days in childcare; previous studies have typically examined lunch only on a single day. However, our study also has limitations. Our sample was small, and it is unlikely representative of all children and centres utilising CACFP. Our estimates are also only one snapshot in time; children were not followed over different seasons or periods of growth. We were unable to measure dietary intake outside of the childcare day, and therefore we cannot determine how foods consumed during this period impact their energy balance over the course of a full day. Finally, the CACFP portion size standards are different than the DGA guidelines used to determine intake, therefore we are unable to directly compare what was served *v*. consumed with the same national guidelines or metric. For example, in the CACFP standards, milk is a separate category, and other dairy foods are included in the meat/meat alternate category; the DGA dairy category includes milk and all dairy foods with a separate protein category for meats. However, given that meals served through CACFP are supposed to support intake that would be adherent to the DGA, and thus the guidelines are meant to be related to one another, it is reasonable to use one set of standards for evaluating food service quality and the other for evaluating consumption quality.

## Conclusions

Our findings suggest sub-optimal intake of DGA recommendations for foods and beverages among children attending childcare, even when centres are compliant with CACFP standards. Childcare is an important space for providing healthy food to children, but this study suggests that childcare meals could be further improved. Further strengthening CACFP minimum standards, supporting providers in implementation stronger nutrition standards, and identifying how to promote children’s intake of CACFP meals may be needed to ensure that children meet DGA recommendations for an optimal healthy diet. National recommendations for dietary intake while in childcare should be considered to ensure that children are consuming adequate amounts of healthy food for optimal growth.

## Supplementary material

For supplementary material accompanying this paper visit https://doi.org/10.1017/S136898002300109X. click here to view supplementary material
