# Supplementary material for: Comparison of foods and beverages served and consumed in Child and Adult Care Food Program-participating childcare centres to national guidelines
Source: Public Health Nutr. 2023 Jun 8;26(9):1862–70. doi: 10.1017/S136898002300109X (PMC10478045; doi:10.1017/S136898002300109X)
Supplement: Supplementary file 1 [file S136898002300109Xsup001.docx]

**Appendix 1. Child and Adult Care Food Program Meal Patterns for ages 3-5 years old; minimum quantities of food components that are required to be served, 2017. ^1,2,3^**

|  | **Minimum quantities of food components** |
| --- | --- |
| **Breakfast Meal Patterns** |  |
| Fruit and/or vegetable | 0.5 cup |
| Grain | 0.5 oz eq |
| Skim or 1% white milk | 0.75 cup |
| Meat/ meat alternates | 0.5 oz eq |
| **Lunch Meal Patterns** |  |
| Fruit | 0.25 cup |
| Vegetable | 0.25 cup |
| Grain | 0.5 oz eq |
| Skim or 1% white milk | 0.75 cup |
| Meat/ meat alternates | 1.5 oz |
| **Snack Meal Patterns ^4^** |  |
| Fruit | 0.5 cup |
| Vegetable | 0.5 cup |
| Grain | 0.5 oz eq |
| Skim or 1% white milk | 0.5 cup |
| Meat/ meat alternates | 0.5 oz |

^1^ Reference: United States Department of Agriculture. Updated Child and Adult Care Food Program Meal Patterns: Child and Adult Meals, 2017. <https://fns-prod.azureedge.net/sites/default/files/cacfp/CACFP_MealBP.pdf> . Accessed 11/04/2021.

^2^ Oz eq= ounce equivalents

^3^ Juice is limited to once per day; at least one serving of grains per day must be whole grain-rich; grain based dessert do not count toward the grain component; yogurt must contain no more than 23 grams of sugar per 6 ounce; and breakfast cereals must contain no more than 6 grams of sugar per dry ounce; Meat and meat alternates may be used to substitute the entire grains component a maximum of three times per week at breakfast. Two vegetables can be offered at lunch in lieu of 1 fruit and 1 vegetable.

^4^ Select 2 of the 5 components for snack.

**Appendix 2: Dietary Guidelines for Americans recommended ranges for calories and food group per day while in childcare, and by eating occasion during child care.^1^**

|  | **DGA recommended amount for child age 2 years** | | | | **DGA recommended amount for moderate active female child age 3 years** | | | | **DGA recommended amount/day for moderately active male age 3 or age 4-5 years** | | | |
| --- | --- | --- | --- | --- | --- | --- | --- | --- | --- | --- | --- | --- |
|  | **Per day** | **During full day child care** | | | **Per day** | **During full day child care** | | | **Per day** | **During full day child care** | | |
|  |  | **Median** | **Minimum standard** | **Maximum standard** |  | **Median** | **Minimum standard** | **Maximum standard** |  | **Median** | **Minimum standard** | **Maximum standard** |
|  |  |  | *0.5* | *0.67* |  |  | *0.5* | *0.67* |  |  | *0.5* | *0.67* |
|  |  | *50% to 67% of recommended daily amount* | | |  | *50% to 67% of recommended daily amount* | | |  | *50% to 67% of recommended daily amount* | | |
| **Consuming breakfast, lunch, and snack** | | | | | | | | | | | | |
| Calories (kJ) | 4184 | 2448 | 2092 | 2803 | 5021 | 2937 | 2510 | 3364 | 5858 | 3427 | 2929 | 3925 |
| Fruit (c) | 1 | 0.59 | 0.50 | 0.67 | 1 | 0.59 | 0.50 | 0.67 | 1.5 | 0.88 | 0.75 | 1.01 |
| Vegetable (c) | 1 | 0.59 | 0.50 | 0.67 | 1.5 | 0.88 | 0.75 | 1.01 | 1.5 | 0.88 | 0.75 | 1.01 |
| Grain (oz) | 3 | 1.76 | 1.50 | 2.01 | 4 | 2.34 | 2.00 | 2.68 | 5 | 2.93 | 2.50 | 3.35 |
| Whole grains | 1.5 | 0.88 | 0.75 | 1.01 | 2 | 1.17 | 1.00 | 1.34 | 2.5 | 1.46 | 1.25 | 1.68 |
| Refined grains | 1.5 | 0.88 | 0.75 | 1.01 | 2 | 1.17 | 1.00 | 1.34 | 2.5 | 1.46 | 1.25 | 1.68 |
| Dairy (c) | 2 | 1.17 | 1.00 | 1.34 | 2.5 | 1.46 | 1.25 | 1.68 | 2.5 | 1.46 | 1.25 | 1.68 |
| Protein (oz) | 2 | 1.17 | 1.00 | 1.34 | 3 | 1.76 | 1.50 | 2.01 | 4 | 2.34 | 2.00 | 2.68 |
|  |  |  | *0.125* | *0.168* |  |  | *0.125* | *0.168* |  |  | *0.125* | *0.168* |
|  |  | *12.5% to 16.8% of recommended daily amount* | | |  | *12.5% to 16.8% of recommended daily amount* | | |  | *12.5% to 16.8% of recommended daily amount* | | |
| **Breakfast** | | | | | | | | | | | | |
| Calories (kJ) |  | 612 | 523 | 701 |  | 734 | 628 | 841 |  | 857 | 732 | 981 |
| Fruit (c) |  | 0.15 | 0.13 | 0.17 |  | 0.15 | 0.13 | 0.17 |  | 0.22 | 0.19 | 0.25 |
| Vegetable (c) |  | 0.15 | 0.13 | 0.17 |  | 0.22 | 0.19 | 0.25 |  | 0.22 | 0.19 | 0.25 |
| Grain (oz) |  | 0.44 | 0.38 | 0.50 |  | 0.59 | 0.50 | 0.67 |  | 0.73 | 0.63 | 0.84 |
| Whole grains |  | 0.22 | 0.19 | 0.25 |  | 0.29 | 0.25 | 0.34 |  | 0.37 | 0.31 | 0.42 |
| Refined grains |  | 0.22 | 0.19 | 0.25 |  | 0.29 | 0.25 | 0.34 |  | 0.37 | 0.31 | 0.42 |
| Dairy (c) |  | 0.29 | 0.25 | 0.34 |  | 0.37 | 0.31 | 0.42 |  | 0.37 | 0.31 | 0.42 |
| Protein (oz) |  | 0.29 | 0.25 | 0.34 |  | 0.44 | 0.38 | 0.50 |  | 0.59 | 0.50 | 0.67 |
|  |  |  | *0.25* | *0.335* |  |  | *0.25* | *0.335* |  |  | *0.25* | *0.335* |
|  |  | *25% to 33.5% of recommended daily amount* | | |  | *25% to 33.5% of recommended daily amount* | | |  | *25% to 33.5% of recommended daily amount* | | |
| **Lunch** | | | | | | | | | | | | |
| Calories (kJ) |  | 1224 | 1046 | 1402 |  | 1469 | 1255 | 1682 |  | 1713 | 1464 | 1962 |
| Fruit (c) |  | 0.29 | 0.25 | 0.34 |  | 0.29 | 0.25 | 0.34 |  | 0.44 | 0.38 | 0.50 |
| Vegetable (c) |  | 0.29 | 0.25 | 0.34 |  | 0.44 | 0.38 | 0.50 |  | 0.44 | 0.38 | 0.50 |
| Grain (oz) |  | 0.88 | 0.75 | 1.01 |  | 1.17 | 1.00 | 1.34 |  | 1.46 | 1.25 | 1.68 |
| Whole grains |  | 0.44 | 0.38 | 0.50 |  | 0.59 | 0.50 | 0.67 |  | 0.73 | 0.63 | 0.84 |
| Refined grains |  | 0.44 | 0.38 | 0.50 |  | 0.59 | 0.50 | 0.67 |  | 0.73 | 0.63 | 0.84 |
| Dairy (c) |  | 0.59 | 0.50 | 0.67 |  | 0.73 | 0.63 | 0.84 |  | 0.73 | 0.63 | 0.84 |
| Protein (oz) |  | 0.59 | 0.50 | 0.67 |  | 0.88 | 0.75 | 1.01 |  | 1.17 | 1.00 | 1.34 |
|  |  |  | *0.125* | *0.168* |  |  | *0.125* | *0.168* |  |  | *0.125* | *0.168* |
|  |  | *12.5% to 16.8% of recommended daily amount* | | |  | *12.5% to 16.8% of recommended daily amount* | | |  | *12.5% to 16.8% of recommended daily amount* | | |
| **Snack** | | | | | | | | | | | | |
| Calories (kJ) |  | 612 | 523 | 701 |  | 734 | 628 | 841 |  | 857 | 732 | 981 |
| Fruit (c) |  | 0.15 | 0.13 | 0.17 |  | 0.15 | 0.13 | 0.17 |  | 0.22 | 0.19 | 0.25 |
| Vegetable (c) |  | 0.15 | 0.13 | 0.17 |  | 0.22 | 0.19 | 0.25 |  | 0.22 | 0.19 | 0.25 |
| Grain (oz) |  | 0.44 | 0.38 | 0.50 |  | 0.59 | 0.50 | 0.67 |  | 0.73 | 0.63 | 0.84 |
| Whole grains |  | 0.22 | 0.19 | 0.25 |  | 0.29 | 0.25 | 0.34 |  | 0.37 | 0.31 | 0.42 |
| Refined grains |  | 0.22 | 0.19 | 0.25 |  | 0.29 | 0.25 | 0.34 |  | 0.37 | 0.31 | 0.42 |
| Dairy (c) |  | 0.29 | 0.25 | 0.34 |  | 0.37 | 0.31 | 0.42 |  | 0.37 | 0.31 | 0.42 |
| Protein (oz) |  | 0.29 | 0.25 | 0.34 |  | 0.44 | 0.38 | 0.50 |  | 0.59 | 0.50 | 0.67 |

^1^ Reference: U.S. Department of Agriculture and U.S. Department of Health and Human Services. Dietary Guidelines for Americans, 2020-2025.

9th Edition. December 2020. Available at DietaryGuidelines.gov. <https://www.dietaryguidelines.gov/sites/default/files/2020-12/Dietary_Guidelines_for_Americans_2020-2025.pdf>

Accessed 11/04/2021

**Appendix 3: Percent of snacks and meals meeting the Child and Adult Care Food Program nutrition standards in six childcare programs in Boston MA, December 2019-March 2020. (N=25 meals observed) ^1,2^**

|  | **N meals meeting CACFP standards** | **Percent meals meeting CACFP standards** |
| --- | --- | --- |
| **Total Meals Observed N=25** | 20 | 80.0 |
| **Breakfast N=7** | 5 | 71.4 |
| Fruit and/or vegetable | 6 | 85.7 |
| Grain | 6 | 85.7 |
| Skim or 1% white milk | 6 | 85.7 |
| Meat/ meat alternates | 4 | 57.1 |
| **Lunch N=10** | 7 | 70.0 |
| Fruit | 8 | 80.0 |
| Vegetable | 10 | 100 |
| Grain | 10 | 100 |
| Skim or 1% white milk | 7 | 70.0 |
| Meat/ meat alternates | 10 | 100 |
| **Snack N=8** | 7 | 87.5 |
| Fruit | 6 | 75.0 |
| Vegetable | 2 | 25.0 |
| Grain | 7 | 87.5 |
| Skim or 1% white milk | 2 | 25.0 |
| Meat/ meat alternates | 4 | 50.0 |
| **Daily requirement N=10** |  |  |
| One whole grain per day | 10 | 100 |
| No grain-based desserts | 9 | 90.0 |
| Juice limited to once per day | 10 | 100 |
| No sugary beverages | 9 | 90.0 |

^1^ Meat and meat alternates may be used to substitute the entire grains component a maximum of three times per week at breakfast. Two vegetables can be offered at lunch in lieu of 1 fruit and 1 vegetable. Select 2 of the 5 components for snack.

^2^ Daily requirement by day; there were 5 observation days with 2 eating occasions and 5 observation days with 3 eating occasions. Juice is limited to once per day; at least one serving of grains per day must be whole grain-rich; grain based dessert do not count toward the grain component; yogurt must contain no more than 23 grams of sugar per 6 ounce; and breakfast cereals must contain no more than 6 grams of sugar per dry ounce. All yogurts and dry cereals served met the sugar limits.
